# Necroptosis mediates myofibre death in dystrophin-deficient mice

**DOI:** 10.1038/s41467-018-06057-9

**Published:** 2018-09-07

**Authors:** Jennifer E. Morgan, Alexandre Prola, Virginie Mariot, Veronica Pini, Jinhong Meng, Christophe Hourde, Julie Dumonceaux, Francesco Conti, Frederic Relaix, Francois-Jerôme Authier, Laurent Tiret, Francesco Muntoni, Maximilien Bencze

**Affiliations:** 1 The Dubowitz Neuromuscular Centre, Molecular Neurosciences Section, Developmental Neurosciences Programme, UCL Great Ormond Street Institute of Child Health, London, WC1N 1EH UK; 2 U955-IMRB, Team 10, Biology of the Neuromuscular System, Inserm, UPEC, ENVA, EFS, Créteil, 94000 France; 3 NIHR Biomedical Research Centre, University College London, Great Ormond Street Institute of Child Health and Great Ormond Street Hospital NHS Trust, 30 Guilford Street, London, WC1N 1EH UK; 4 Inter-University Laboratory of Human Movement Biology (LIBM)-EA7424, Université Savoie Mont Blanc, Campus Scientifique Technolac, 73376 Le Bourget du Lac Cedex, France; 5 Nord/Est/Ile-de-France Reference Centre for Neuromuscular Diseases, Henri Mondor University Hospital (APHP), 94000 Créteil, France

## Abstract

Duchenne muscular dystrophy (DMD) is a severe degenerative disorder caused by mutations in the dystrophin gene. Dystrophin-deficient muscles are characterised by progressive myofibre necrosis in which inflammation plays a deleterious role. However, the molecular mechanisms underlying inflammation-induced necrosis in muscle cells are unknown. Here we show that necroptosis is a mechanism underlying myofibre death in dystrophin-deficient muscle. RIPK1, RIPK3 and MLKL are upregulated in dystrophic mouse myofibres. In human DMD samples, there is strong immunoreactivity to RIPK3 and phospho-MLKL in myofibres. In vitro, TNFα can elicit necroptosis in C2C12 myoblasts, and RIPK3 overexpression sensitises myoblasts to undergo TNF-induced death. Furthermore, genetic ablation of *Ripk3* in mdx mice reduces myofibre degeneration, inflammatory infiltrate, and muscle fibrosis, and eventually improves muscle function. These findings provide the first evidence of necroptotic cell death in a disease affecting skeletal muscle and identify RIPK3 as a key player in the degenerative process in dystrophin-deficient muscles.

## Abstract (web summary)

Muscular dystrophies are characterised by extensive myofibre cell death. Here Morgan et al. show that RIPK3-mediated necroptosis contributes to myofibre cell death in Duchenne muscular dystrophy, and that RIPK3 deletion protects dystrophic mice against myofibre degeneration.

## Introduction

The progressive degeneration of muscle fibres (myofibres) is a hallmark of many neuromuscular disorders, including Duchenne muscular dystrophy (DMD)^1^. DMD is caused by mutations in the dystrophin gene and affects 1 in 5000 male births^2^. While its pathogenesis has been extensively investigated, the molecular basis of cell death affecting dystrophin-deficient myofibres remains elusive. The involvement of apoptosis has been proposed because nuclei with apoptotic DNA fragmentation are found in muscles of DMD patients and of the C57BL/10ScSn-*Dmd*^*mdx*^ (hereafter named mdx) mouse model of DMD^3–5^. However, it is relatively rare, suggesting a limited contribution of apoptotic death in the loss of muscle fibres. In contrast, a necrotic morphology characterises most degenerating myofibres in DMD^5,6^.

The induction of cell death is multifactorial: dystrophin deficiency renders myofibres more susceptible to mechanical stress, and cytotoxic factors induced by the inflammatory process participate in muscle loss^7,8^. Although molecular triggers and cytosolic death pathway(s) in myofibres necrosis remain elusive, the tumour necrosis factor-α (TNFα) pro-inflammatory cytokine has a strong pro-necrotic effect in mdx myofibres^9,10^.

Recently, a genetically controlled form of necrosis named necroptosis has been identified^11^. Although necroptosis is a caspase-independent mechanism, its signalling partially overlaps with extrinsic apoptosis. These two forms of programmed cell death can be triggered by TNF receptor superfamily ligands, including TNFα. Necroptosis often requires receptor interacting protein kinase-1 (RIPK1) activity in caspase-compromised conditions, and it critically depends on RIPK3 and mixed lineage kinase domain like pseudokinase (MLKL)^12^. Furthermore, necroptosis is involved in several pathogenic processes affecting solid organs, including ischaemia/reperfusion of the brain and heart^13^. The pathophysiological relevance of necroptosis in skeletal muscle degeneration remains to be established.

Here, we show that necroptosis contributes to myofibre death in dystrophin-deficient skeletal muscle. We demonstrate that TNFα can trigger caspase-independent cell death in myogenic cells, by activating RIPK3-dependent necroptotic signalling. We find evidence of necroptosis in degenerating dystrophic muscles from DMD patients and dystrophin-deficient mdx mice, associated with RIPK3 upregulation. Importantly, mdx mice deficient in RIPK3 had reduced myofibre necrosis and muscle fibrosis and present with improvement in muscle function. This study provides evidence of programmed necrosis in a pathology affecting skeletal muscle, highlighting the relevance of necroptotic cell death to DMD pathogenesis.

## Results

### Necroptosis is activated in dystrophin-deficient mouse and human muscles

**Fig. 1:**
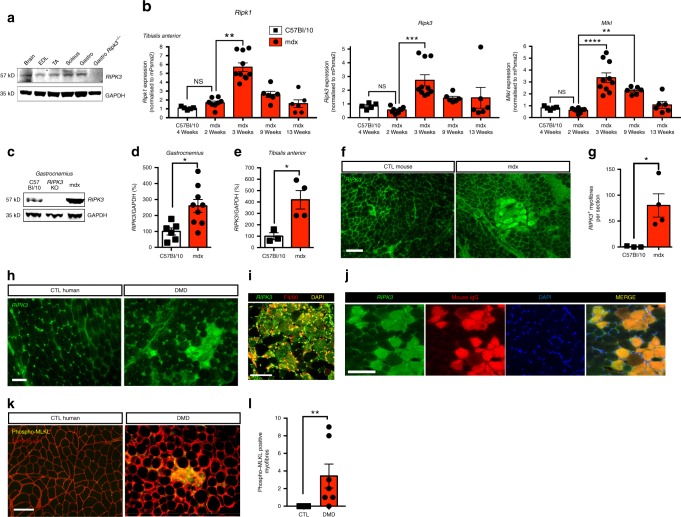
Necroptosis is activated in mouse and human dystrophin-deficient muscles. **a** Immunoblot of RIPK3 protein expression in brain, *extensor digitorum longus* (EDL), *tibialis anterior* (TA), *soleus*, and *gastrocnemius* muscles of C57BL/6 and *gastrocnemius* from RIPK3 KO mouse. GAPDH was used as loading control. **b** *Tibialis anterior* muscles of 4-week-old C57BL/10, and 2-, 3-, 9-, and 13-week-old mdx mice were analysed for *Ripk1*, *Ripk3*, and *Mlkl* mRNA levels by quantitative PCR. Data were normalised to mouse *Psma2* gene expression (*n* = 5 C57BL/10 and *n* = 5, 8, 10, 6, and 6 respectively, for 2-, 3-, 9-, and 13-week mdx TAs, Dunn’s multiple comparison test). **c** Protein extracts of C57BL/10, RIPK3 KO, and mdx *gastrocnemius* muscles were analysed by western blot for RIPK3 and GAPDH protein expression. Quantification of RIPK3 protein expression normalised to GAPDH in *gastrocnemius* (**d**) and TA (**e**) (*n* = 6 C57BL/10 and *n* = 9 mdx *gastrocnemius;* *n* = 3 C57BL/10 and *n* = 5 mdx TA, Student’s *t*-test). **f** Mouse *gastrocnemius* C57BL10 (left) and mdx (right) mice were immunolabelled with an antibody to RIPK3 (green). **g** Quantification of RIPK3-positive myofibres in *gastrocnemius* of C57BL10 (*n* = 3) and mdx mice (*n* = 4). **h** Representative transverse sections of quadriceps biopsies from a healthy non-dystrophic (CTL human) and a DMD patient, immunolabelled with an antibody to RIPK3 (green). **i** Representative image of immunostaining using antibodies to RIPK3 (green) and mouse IgG (red), a functional marker for membrane permeability in myofibres. **j** Representative image of immunostaining using antibodies to RIPK3 (green) and F4/80 (red), a marker for murine macrophages. **k** Confocal image of transverse cryosections of DMD quadriceps labelled with antibodies to human phospho-MLKL (green), human laminin α2 (red), and DAPI (blue). **l** Quantification of the number of p-MLKL-positive myofibres per field (*n* = 5 control human and *n* = 6 DMD). Data presented as the mean ± SEM. Scale bars, 100 µm. *P < 0.05, **P < 0.01, ***P < 0.001, ****P < 0.0001

RIPK3 expression is essential for the induction of canonical necroptosis^14,15^. To determine whether skeletal muscle is necroptosis competent, we initially examined the levels of RIPK3 in normal hindlimb muscles of C57BL/6 mice by western blot. RIPK3 protein was present in *extensor digitorum longus* (EDL), *soleus*, *gastrocnemius*, and *tibialis anterior* (TA) muscles of adult mice (Fig. 1a). Levels of RIPK3 in hindlimb muscles were comparable to those found in the brain (Fig. 1a), a well-established necroptotic-competent tissue^11^.

Next, we investigated the presence of necroptosis in dystrophin-deficient muscles. The upregulation of proteins belonging to the necroptosis machinery (i.e., RIPK1, MLKL, and more specifically RIPK3) is a strong indication of necroptosis in vivo^16^. The extent of myofibre degeneration in hindlimb muscles of mdx mice varies over the lifespan: muscle fibre degeneration begins around weaning age at 3 to 4 postnatal weeks, and then decreases^17,18^. Therefore, we evaluated the levels of necroptosis biomarkers in TA muscles from mdx mice of different ages by quantitative PCR (Fig. 1b). In TAs of 2-week-old mdx mice (i.e., before the onset of myonecrosis), the levels of *Ripk1*, *Ripk3*, and *Mlkl* transcripts were similar to those in TAs from C57BL/10 controls. At 21 days (corresponding to the degenerative spike in TA muscles), *Ripk1*, *Ripk3*, and *Mlkl* transcripts dramatically increased (Dunn’s multiple comparison tests, *Ripk1*: *P* = 0.0022, *Ripk3*: *P* *<* 0.0001, *Mlkl*: *P* *<* 0.0001). All transcripts were decreased at 9 and 13 weeks compared to 3 weeks, suggesting a transitory necroptotic peak at 3 weeks in TA muscle (Fig. 1b). Nevertheless, *Mlkl* transcript remained threefold higher at 9 weeks than at 2 weeks (*P* = 0.0017) in TA muscles. In 3–4-week-old mdx mice, RIPK3 upregulation at the protein level was confirmed in TA and *gastrocnemius* muscles (Fig. 1c–e).

We then examined the localisation profile of RIPK3 within dystrophic muscles by immunolabelling. In dystrophic muscles from mdx mice or DMD patients, we observed areas with strong sarcoplasmic immunoreactivity to RIPK3 antibody, which were not seen in mouse or human controls (Fig. 1f–h). This may reflect local RIPK3 overexpression at the myofibre level. Next, we examined the profile of RIPK3-immunoreactive myofibres and found F4/80-positive macrophages surrounding them, suggesting the recruitment of phagocyte cells. High RIPK3 immunoreactivity in mdx myofibres was also strongly associated with mouse immunoglobulin G (IgG) uptake, a marker for necrosis in muscle tissue^19^ (Fig. 1g).

RIPK3 immunolabelling was performed using a RIPK3 antibody raised in rabbit. Its immunoreactivity was due to the binding of the primary antibody and not to the binding of the secondary antibody (Supplementary Fig. 1). Overall, the correlation of RIPK3 upregulation with necrotic cell fate may also suggest a RIPK3-dependent death mechanism.

In order to confirm the significance of these findings in human pathology, we labelled quadriceps samples of DMD patients with an antibody directed against human phospho-MLKL, which is the only available marker for necroptosis^20^. We observed MLKL-positive myofibres in DMD samples but not in control patients (Fig. 1k–l), indicating the activation of the final step of the necroptotic pathway by some DMD muscle fibres.

Together, these data suggest that the necroptotic machinery is activated in degenerating dystrophin-deficient muscles.

### RIPK3 upregulation sensitises TNFα-induced necroptosis in C2C12 myoblasts

**Fig. 2:**
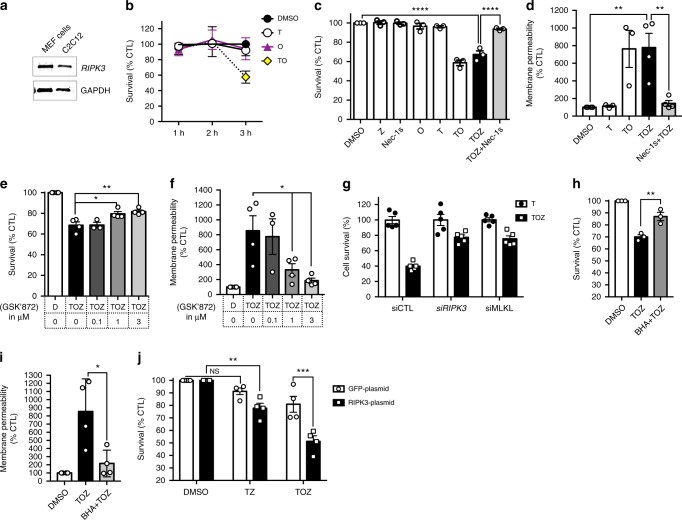
RIPK3 upregulation facilitates TNFα-induced necroptosis in C2C12 myoblasts. **a** Immunoblot of RIPK3 expression in mouse embryonic fibroblast (MEF) cells and C2C12 myoblasts. **b** C2C12 myoblasts were exposed for 1, 2, or 3 h to either DMSO, mouse TNFα (100 ng/ml), TAK1 inhibitor (5Z)-7-Oxozeaenol (1 µM), and TNFα+TAK1 inhibitor (respectively T, O, and TO treatment) and cell survival was assessed by measuring ATP levels (CellTiter-Glo assay). Levels of ATP in DMSO-treated cells were defined as 100% survival. Data represented as the mean ± SEM of two independent experiments. **c** C2C12 myoblasts were challenged for 3 h with TNFα, (5Z)-7-Oxozeaenol, the pan-caspase inhibitor Z-VAD.fmk at 50 µM (Z), and the inhibitor of RIPK1 kinase activity Necrostatin-1s (Nec-1s) at 30 µM. Cell survival was assessed. Data presented as the mean ± SEM of three pooled independent experiments (*n* = 3, one-way ANOVA). **d** Same experimental conditions as in (**c**). Membrane permeability of treated cells was assessed using CytoTox-Glo assay after 3 h of treatment. Data represented as the mean ± SEM of three pooled independent experiments (*n* = 3, one-way ANOVA). **e** Cells were treated with TOZ for 3 h, with GSK’872, a RIPK3 inhibitor. Cell survival was measured. Data presented as the mean ± SEM of four pooled independent experiments (*n* = 4, one-way ANOVA). **f** Cells were treated as in **e** and membrane permeability was monitored by CytoTox-Glo assay. Data presented as the mean ± SEM of four pooled independent experiments (*n* = 4, one-way ANOVA). **g** *Ripk*3 or *Mlkl* mRNAs were knocked down using specific RNAi. Transfected C2C12 cells were treated with either TNFα alone or TOZ and cell survival was assessed. Representative of two independent experiments (five replicates per group). **h**–**i** C2C12 myoblasts were challenged by TOZ with or without Butylated hydroxyanisole (BHA). Cell survival (**h**) and membrane permeability (**i**) were monitored. Data presented as the mean ± SEM of four pooled independent experiments (*n* = 4, one-way ANOVA). **j** C2C12 myoblasts were transfected with a *GFP* plasmid or a *GFP*-tagged *Ripk3* plasmid. Transfected cells were stimulated and the survival was monitored (*n* = 4 pooled independent experiments, two-way ANOVA). Data shown as the mean ± SEM. *P < 0.05, **P < 0.01, ***P < 0.001, ****P < 0.0001

To elucidate the signalling events underlying necroptosis in skeletal muscle cells, we investigated necroptosis induction in myogenic cells. C2C12 myoblasts express significant levels of RIPK3 and are shown with mouse embryonic fibroblasts (MEFs), which served as positive control (Fig. 2a). Since TNFα is pro-necrotic in mdx mice^9,10^, and is also a well-established necroptotic trigger in cardiomyocytes^12,21^, we used TNFα to induce necrotic demise in myoblasts.

TNFα stimulation on its own failed to reduce cellular adenosine triphosphate (ATP) content in C2C12 myoblasts (Fig. 2b). Indeed, eliciting cell death by TNFα treatment often requires sensitising methods, such as the pharmacological inhibition of transforming growth factor-beta-activated kinase-1 (TAK1), which has pro-survival activity following TNFα binding^22^. Therefore, we used (5Z)-7-Oxozeaenol, a TAK1 inhibitor (O treatment), to sensitise cells to TNF-induced cell death. TAK1 inhibition alone did not affect cell survival (Fig. 2b–d). However, when combined with TNFα stimulation (TO treatment), intracellular ATP levels were reduced by around 40% within 3 h (Fig. 2b, c). ATP decrease in C2C12 myoblasts was associated with a markedly (~700%) increase of their membrane permeability (Fig. 2d). Taken together, ATP reduction and the induction of plasma membrane permeability in C2C12 cells are indicative of cell death.

To exclude the involvement of apoptosis in the death induced by TO treatment, we blocked caspases using Z-VAD.fmk, a pan-caspase inhibitor (TOZ treatment), which did not significantly restore initial levels of ATP (Fig. 2c) or plasma membrane integrity (Fig. 2d). Death elicited by TOZ treatment was rescued by necrostatin-1s (Nec-1s), an inhibitor of RIPK1 kinase activity^11,23^(Fig. 2c, d). These data demonstrate that TNFα can trigger a caspase-independent cell death involving RIPK1 activity in C2C12 myoblasts, and suggest activation of the early steps of the necroptotic pathway^24^. To further support the involvement of necroptosis in TNF-induced myoblast death, we either pharmacologically inhibited RIPK3 activity in a dose-dependent manner with the RIPK3 inhibitor GSK’872^25^ (Fig. 2e, f), or silenced *Ripk*3 and *Mlkl* expression by RNA interference (RNAi; Fig. 2g, Supplementary Fig. 1a,b). All treatments reduced C2C12 death elicited by TOZ treatment, confirming that TNFα can elicit necrosis via the necroptotic pathway in myoblasts. Moreover, it is well established that reactive oxygen species (ROS) generation can participate in necroptotic death execution^26,27^. Interestingly, we found that the ROS scavenger butylated hydroxyanisole (BHA) protected myoblasts against necroptosis (Fig. 2h, i), suggesting that oxidative stress also participates in the execution of TNF-induced necroptosis in C2C12 myoblasts.

To relate these findings to dystrophic pathology, we investigated under which conditions TNFα can induce necroptosis without sensitising methods such as TAK1 inhibition. Since the overexpression of RIPK3 potentiates TNF-induced necroptosis in cardiomyocytes^21^, we transfected C2C12 cells with green fluorescent protein (GFP)-tagged or *Ripk3 GFP*-tagged plasmids^28^ (Supplementary Fig. 2c). Transfected cells were GFP-sorted and then stimulated with necrotic triggers. Cell death induced by TOZ treatment was greater in C2C12 cells overexpressing RIPK3 (Fig. 2j). Importantly, we found that TNFα stimulation in caspase-compromised conditions, without TAK1 inhibition, could induce cell death in myoblasts overexpressing RIPK3 (TZ treatment). This indicates that high RIPK3 expression is sufficient to facilitate TNF-induced necroptosis in myoblasts.

### RIPK3 depletion alleviates muscle necrosis in mdx mice

Having demonstrated that muscle cells can undergo RIPK1-, RIPK3-, and MLKL-dependent necrosis in vitro, and that necroptosis occurs in dystrophin-deficient muscles, we aimed to determine the contribution of necroptosis to mdx myofibre death. We crossed RIPK3 knockout (KO) with mdx mice. Overall, *Ripk3*^*−/−*^ mice have a normal phenotype when unchallenged, but are more resistant to cell death induced by necroptotic stimuli in various tissues^29^. Nonetheless, the effect of RIPK3 deficiency in skeletal muscle degeneration has never been reported. mdx*Ripk3*^*+/+*^ (mdx) and mdx*Ripk3*^*−/−*^ littermates were generated by intercrossing mdx*Ripk3*^*+/−*^ mice. No difference was observed in overall body weight, nor in the mass of isolated TA muscles between mdx*Ripk3*^*+/+*^ (mdx), mdx*Ripk3*^*+/−*^ and mdx*Ripk3*^*−/−*^ littermates in adult mice (Supplementary Fig. 3a-c).

**Fig. 3:**
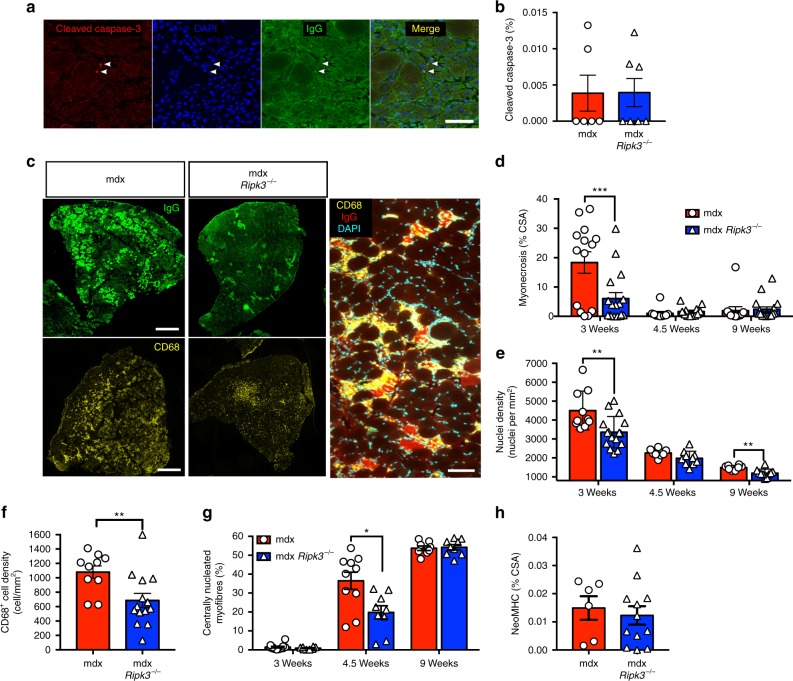
RIPK3 deficiency reduces the acute phase of myonecrosis in mdx mice. **a** Representative images of cleaved caspase-3 immunolabelling in 3-week-old mdx*Ripk3*^*−/−*^ TA. Scale bar, 50 µm. **b** Quantification of the percentage of cleaved caspase-3-positive nuclei. **c** Representative images of IgG uptake and CD68-positive cells in mdx (mdx*Ripk3*^+/+^) and mdx*Ripk3*^*−/−*^ muscles. Scale bars, 500 µm (left panels) and 50 µm (right panel). **d** Quantification of the extent of myonecrosis with age in mdx and mdx*Ripk3*^*−/−*^ littermates. Animals were analysed at 3 weeks (*n* = 14 mdx*Ripk3*^+/+^muscles, *n* = 17 mdx*Ripk3*^*−/−*^ muscles), 4.5 weeks (*n* = 10 mdx, *n* = 12 mdx*Ripk3*^*−/−*^), and 9 weeks of age (*n* = 12 mdx, *n* = 18 mdx*Ripk3*^*−/−*^). The extent of myonecrosis was defined as the area of IgG uptake in myofibres, and expressed as percentage of the cross-sectional area (%CSA). Two-way ANOVA, Sidak’s multiple comparisons test. **e** Quantification of nuclear density in TA muscles at 3 weeks (*n* = 10 mdx, *n* = 16 mdx*Ripk3*^*−/−*^*)*, 4.5 weeks, and 9 weeks (*n* = 8 mdx, *n* = 10 mdx*Ripk3*^*−/−*^). **f** Quantification of CD68-positive cell infiltration in TA of 3-week-old mice (*n* = 10 mdx*Ripk3*^+/+,^*n* = 12 mdx*Ripk3*^*−/−*^ muscles, Student’s *t*-test). **f** Quantification of neoMHC staining in 4.5-week-old mice. Data expressed as percentage of cross-section area (*n* = 6 mdx*Ripk3*+/+ and *n* = 12 mdx*Ripk3*^*−/−*^ TA). **g** Quantification of the percentage of centrally nucleated fibres (*n* = 8 mdx*Ripk3*^+/+^ and *n* = 9 mdx*Ripk3*^*−/−*^ TA). **h** Quantification of NeoMHC-positive myofibres at 4.5 weeks (% of CSA). Data presented as the mean ± SEM. *P < 0.05, **P < 0.01, ***P < 0.001

Because apoptosis and necroptosis are interconnected death pathways, we checked whether the inhibition of RIPK3-dependent necroptosis would promote apoptosis in mdx muscles. Since the TUNEL (terminal deoxynucleotidyl transferase dUTP nick end labeling) assay is known to be an unreliable method for labelling apoptotic death^30^, muscles of mdx and mdx*Ripk3*^*−/−*^ mice were instead analysed using cleaved caspase-3 antibody (Fig. 3a, b). Very rare positive cells were found in both groups (around 0.004% of total nuclei). Of note, no positive nuclei were observed within the myofibre sarcolemma (Fig. 3a). This indicates that RIPK3 deficiency does not favour apoptosis in the dystrophin-deficient muscles.

Then, we examined the effect of RIPK3 depletion on mdx muscle degeneration (Fig. 3c, d). Quantifying muscle necrosis at different ages, we found that control mdx mice presented the characteristic necrotic peak at 3 weeks of age (18% of cross-section area (CSA)) followed by lower necrosis at 4.5 and 9 weeks of age (1–2% of CSA)^18^. In mdx*Ripk3*^*−/−*^ mice, we found a threefold reduction in the extent of myofibre necrosis at 3 weeks (2-way analysis of variance (ANOVA), Sidak’s multiple comparisons test, *P* < 0.0002, Fig. 3c, d). Interestingly, mdx*Ripk3*^*−/−*^ mice had statistically similar levels of myofibre death at all tested time points (3 weeks versus 4.5 weeks, *P* = 0.8434; 3 weeks versus 9 weeks, *P* = 0.9916) (Fig. 3b). Muscle necrosis is a powerful inflammatory activator and is promptly followed by macrophage infiltration^31^. Any protection against myofibre death is therefore expected to reduce the postinjury inflammatory infiltrate. IgG uptake reduction in the mdx*Ripk3*^*−/−*^ myofibres was associated with a significant diminution of cell infiltrate (Fig. 3e), and a 40% decrease in macrophages (Fig. 3f), indicating that RIPK3 has a role in the inflammatory response to myonecrosis. Of note, although we found no difference in the quantification of necrotic myofibres at 9 weeks, there was a modest but significant reduction of cell infiltrate in mdx*Ripk3*^*−/−*^ muscles (Fig. 3d, e).

The kinetics of muscle regeneration were established by quantifying the percentage of centrally nucleated myofibres. In both groups, a low and equal percentage of centrally nucleated fibres were found at 3 weeks, indicating that no significant death events occur before weaning. At 4.5 weeks, there was a 40% reduction in centrally nucleated myofibres in mdx*Ripk3*^*−/−*^ muscles compared to mdx*Ripk3*^+/+^ littermates (Fig. 3g). The decrease in the formation of myofibres at 4.5 weeks is in line with the reduction of myonecrosis observed earlier at 3 weeks (Fig. 3d). At 9 weeks, the proportion of central nucleation was similar in both groups and did not support a clear involvement of RIPK3 depletion in the myonecrosis affecting mdx TA muscles in adulthood.

Next we questioned whether RIPK3 depletion affects the basal regenerative capacity of dystrophic muscles. Having observed at 4.5 weeks of age an equal amount of myonecrosis (Fig. 3d) and cell infiltrate (Fig. 3e), we analysed TA muscles using the neonatal myosin heavy chain (NeoMHC) antibody, so that only very recently regenerating myofibres are labelled. The same proportion of neonatal NeoMHC-positive fibres were found in mdx and mdx*Ripk3*^*−/−*^. This suggests that, at comparable injury extent, regeneration is similar.

Together, our data indicate that RIPK3 drives the initial acute phase of muscle degeneration occurring at 3 weeks of age in mdx hindlimb muscles.

### RIPK3 deficiency ameliorates muscle pathology in adult mdx mice

**Fig. 4:**
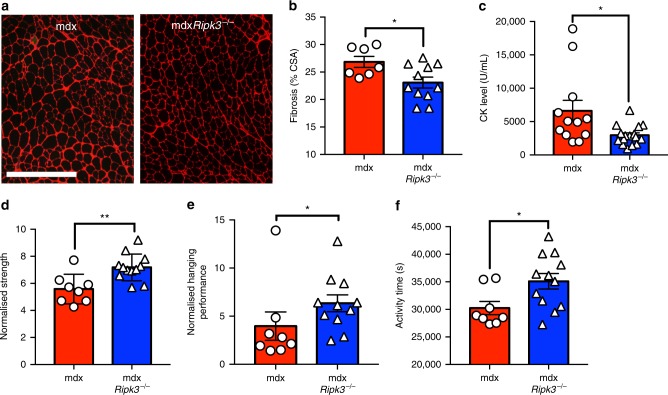
RIPK3 deficiency ameliorates muscle phenotype in adult mice. **a** Representative image of extracellular matrix deposition in TA muscles of 9-week-old mice. Extracellular matrix was stained with antibodies to laminin and collagen VI together (red). Scale bar, 500 µm. **b** Quantification of extracellular matrix deposition (fibrosis) in TA of 9-week-old mice. Total extracellular matrix area was expressed as percentage of cross-sectional area (*n* = 7 mdx, *n* = 11 mdx*Ripk3*^*−/−*^, Student’s *t-*test). **c** Quantification of serum CK in 3-month-old mdx mice (*n* = 13 mdx, *n* = 17 mdx*Ripk3*^*−/−*^, Mann–Whitney test). **d** Quantification of grip strength and **e** hanging performance (expressed as the ratio: maximum hanging time (in s)/body weight; *n* = 8 mdx, *n* = 12 mdx*Ripk3*^*−/−*^, Mann–Whitney test), **f** Quantification of spontaneous activity of mice in 12 h time. Data presented as the mean ± SEM. *P < 0.05, **P < 0.01

We next extended the analysis of the phenotype to adult mdxRIPK3-deficient mice. The pathological occurrence of degeneration/regeneration cycles in DMD and in mdx mice muscles leads to the progressive development of fibrotic tissue. Extracellular matrix deposition was quantified in TA of 9-week-old mice. Mdx*Ripk3*^*−/−*^ mice had reduced muscle fibrosis (Fig. 4a, b). The compensatory hypertrophy of myofibres, which is characteristic of mdx muscles, was also slightly decreased in mdx*Ripk3*^*−/−*^ males but not in females (Supplementary Fig. 4). Furthermore, mdx*Ripk3*^*−/−*^ mice had over 50% diminution in serum creatine kinase (CK) levels (*P* = 0.0150) (Fig. 4c), indicating a broad effect of RIPK3 on the mdx muscle degeneration in adulthood.

Finally, we examined whether the RIPK3-dependent necroptosis ultimately contributes to long-term loss of motor function in mdx mice. RIPK3 deficiency improved the grip strength in adult mdx mice by 20% (*P* = 0.0073) (Fig. 4d). Furthermore, hanging performance was also increased by 30% (*P* = 0.0259) (Fig. 4e). To determine whether the amelioration of muscle function caused by RIPK3 deficiency also influenced their global movement capacities, we assessed the nocturnal spontaneous activity of mice. During the night, mdx*Ripk3*^*−/−*^ mice were more active compared to control mdx mice (*P* = 0.0252) (Fig. 4f), with a substantial although non-statistically significant improvement in the time spent performing fast movements and in the global distance they covered during the night (Supplementary Fig. 5).

Together, these data indicated that RIPK3 has long-term modulatory effects on mdx pathogenesis.

## Discussion

Necroptosis is involved in the pathogenesis of disorders which can affect tissues including brain, heart, liver, kidney, and pancreas^32^. More recently, the necroptotic machinery has also been shown to participate in the degeneration of nucleus pulposus cells^33^, ageing of male reproductive system^34^, and ventilator-induced lung injury^35^. Skeletal muscle is a tissue that alone constitutes 30 to 38% of body mass^36^. In this report, we provide for the first time evidence that necroptosis is involved in a skeletal muscle pathology, more specifically in the degeneration of dystrophin-deficient muscles.

Although the morphology of degenerating myofibres in dystrophin-deficient muscle is described as necrotic, TUNEL-positive nuclei were observed in mdx and DMD muscle samples, suggesting activation of apoptosis^3–5,37^. However, DNA fragmentation labelled by the TUNEL assay is not considered a reliable method to identify apoptotic cell death in situ^30^. Of note, it can also label necroptotic cells^38^. Caspase activity also correlates with dystrophic muscle degeneration^39^. However, caspases also have nonapoptotic roles. More specifically, caspase-3 has been shown to be involved in the cell differentiation process in multiple cell types^40^, including myogenic progenitor differentiation and fusion^41^. Therefore, it cannot be excluded that caspase activation observed in dystrophic muscles is due to myogenic differentiation rather than caspase-dependent cell demise.

Using an antibody directed against the cleaved form of caspase-3, we quantified the number of positive nuclei and found extremely rare events (0.004%) (Fig. 3a, b). Since there was no increase in the proportion of cleaved caspase-3 nuclei in mdx*Ripk3*^*−/−*^ degenerating myofibres (Fig. 3a, b), we concluded that RIPK3 deficiency does not generate a switch from necrotic towards the apoptotic death pathway in mdx muscles. Interestingly, we observed no cleaved caspase-3-positive nuclei within the sarcolemma and so have no evidence of apoptotic demise of myofibres.

Evidence of necroptosis was collected in both mouse and human dystrophin-deficient muscles. Marked elevation of RIPK1, MLKL, and especially RIPK3 is a feature of necroptotic injuries in vivo^14,16,21,42^. We found upregulated expression of these three genes in mdx muscles (Fig. 1b–e). Although macrophages also express necrosome proteins, and their infiltration of necrotic tissue could contribute to the elevation of necroptosis markers in mdx muscles, we showed a strong RIPK3 immunoreactivity within mdx muscle fibres (Fig. 1f, g). These results are in line with the profile of RIPK3 immunoreactivity observed in the necroptotic heart or retina^43,44^. In addition, RIPK3-positive myofibres are associated with a necrotic fate (Fig. 1i, j). In some liver injuries^20,45^, or in the white matter in patients affected by multiple sclerosis^46^, the presence of phospho-MLKL-positive cells is indicative of necroptotic events. Upregulation of *Mlkl* in TA of 3- and 9-week-old mdx and the presence of phospho-MLKL-positive myofibres in DMD muscles (Fig. 1k, l) further indicates necroptosis activation in human dystrophin deficiency.

Historically, inhibition of necroptosis was achieved using the RIPK1 inhibitor necrostatin-1^11^. However, RIPK1-independent forms of necroptosis have been identified^26^. Furthermore, RIPK1 inhibitors have a very short half-life in vivo^11^ making their delivery unsuitable for studying chronic and unsynchronised cell death such as in dystrophin-deficient muscles. Analysis of the necrosome components expression over time showed that *Ripk1*, *Ripk3*, and *Mlkl* fades in mdx TA after the degeneration peak and indicates a transitory period of necroptosis at the onset of the pathology. It is in line with the lack of myonecrosis protection observed in this muscle at 4.5 and 9 weeks (Fig. 3d). To note, *Mlkl* transcript is still upregulated in mdx TA at 9 weeks (Fig. 1b). Also, there is a significant decrease in cell infiltrate at the same time point in mdx*Ripk3*^*−/−*^ muscles (Fig. 3f). This may suggest an existing, but mild, contribution of RIPK3-dependent cell death after the weaning period (Fig. 3d, e) in mdx hindlimb muscles.

Considering the transient activation of necroptosis we observed in mdx TA, we carefully investigated whether RIPK3-dependent necrosis has long-term effect on the overall mdx mouse pathology.

The assessment of the general myonecrosis in adult mdx mice was performed by the quantification of serum CK levels. We found more than 50% decrease of CK levels in 3-month-old mdx*Ripk3*^*−/−*^ mice (Fig. 4c), demonstrating that some muscle groups undergo significant amounts of RIPK3-dependent necrosis in adulthood. Whether respiratory muscles such as the diaphragm, which are more affected by myonecrosis in older mice^47^, undergo more necroptosis than limb muscles will have to be verified in future studies. We further demonstrate the involvement of RIPK3 in the pathogenesis of dystrophin-deficient mice by a protective effect of RIPK3 deficiency on the excessive development of interstitial fibrosis. Indeed, unresolved inflammation linked to chronic degeneration/regeneration episodes and the ultimate exhaustion of the regenerative capacity are a major driver of fibrotic tissue deposition^48^, and is a key factor in the loss of function in DMD patients. The fibrosis reduction in mdx*Ripk3*^*−/−*^ muscles (Fig. 4a, b) is in line with the reduced necrosis and inflammation occurring at earlier time points (Fig. 3c–f).

The functional analysis of adult mdx*Ripk3*^*−/−*^ mice showed a greater grip strength than mdx littermates (Fig. 4d), and was indicative of an improvement of muscle function, with benefits at the organism level revealed by an increased nocturnal feeding, grooming, and locomotor activity (Fig. 4f). Although we observed an improvement in the time spent in fast locomotion and in the total distance covered by mdx*Ripk3*^*−/−*^ mice, it did not reach statistical significance (Supplementary Fig. 5). This may be due to heterogeneity in the mouse phenotype, and is in line with the mild locomotor phenotype observed in mdx mice^49^.

Together, our data demonstrate that RIPK3 is involved in the myonecrosis of dystrophin-deficient mice at all investigated ages, but more specifically at the onset of the pathogenesis in TA muscle. In adulthood, RIPK3 depletion meaningfully improves muscle homoeostasis and function in mdx mice.

Whether RIPK3 deficiency in myeloid cells can participate in the mdx*Ripk3*^*−/−*^ phenotype remains a possibility. Indeed, recent studies suggest a complex and poorly understood role for RIPK3 in the control of inflammation. Lipopolysaccharide can promote a pro-inflammatory response in macrophages via RIPK3, but independently of cell death^50^. Alternatively, necroptosis of myeloid cells is an effective means to halt the production of TNFα and pro-inflammatory cytokines^51^. Therefore, RIPK3 deficiency in myeloid cells could have both pro- or anti-inflammatory effects. Further investigation will require phenotypic analyses of mdx mice with a macrophage-specific genetic inactivation of *Ripk3*.

Our study addresses the questions of the asynchrony and the heterogeneity of muscle cell necrosis in dystrophic mice. Interestingly, while two-thirds of myonecrosis affecting mdx hindlimb muscles at 3 postnatal weeks is driven by RIPK3 (Fig. 3d), our results suggest no crucial involvement of RIPK3 at time points when the TA is undergoing less degeneration later in life. Indeed, the number of TA myofibres which are affected by leaky sarcolemma (trapping serum proteins and IgG) did not significantly vary in *Ripk3*^*+/+*^ and *Ripk3*^*−/−*^ mdx mice at 4.5 and 9 weeks. This indicates that RIPK3 is a major driver of limb muscle degeneration at the onset of mdx pathogenesis in hindlimb muscles, with long-term functional consequences. Since serum CK levels in DMD patients are extremely high in very young patients and gradually decrease with the reduction of locomotor activity in muscle mass^52^, a potential preeminent role of RIPK3 in myonecrosis affecting the very onset of human pathology can be envisaged. Whether a compensatory mechanism fading the necroptosis pathway would occur in dystrophic mice and not in dystrophic humans (and would therefore contribute to the discrepancy between mouse and human pathology) is not to be excluded.

The next important question to address is the identification of death triggers involved in the necroptosis of dystrophic myofibres in vivo. Indeed, factors participating in muscle injury in the mdx mouse, such as inflammatory infiltrate, TNFα, ischaemic stress, or ROS, are also associated at different levels with necroptotic demise in other tissues^8,12,53^. Following muscle injury, neutrophils and M1 macrophages rapidly invade tissue and generate a strong pro-inflammatory environment^31^. The cocktail of pro-inflammatory cytokines secreted by M1 macrophages have powerful cytotoxic effects, but also support satellite cell proliferation, and so they can participate in muscle regeneration^54,55^. High levels of TNFα are released by macrophages and myogenic cells during muscle degeneration, more specifically between 2 to 4 weeks of age in mdx mice^56^, and promote necrosis^9,10^. Our data demonstrate that TNFα is a death ligand that can trigger necroptosis in myoblasts (Fig. 2). While TNFα drives the apoptosis of mdx fibro/adipogenic progenitors^57^, no death mechanism triggered by inflammatory stimuli and leading to necrosis was identified in muscle tissue.

RIPK3 overexpression in hindlimb muscles at 3 weeks, in synergy with the strong TNFα presence in the muscle environment, could provide appropriate conditions to induce myofibre necroptosis. Generally, TNFα is not sufficient to trigger cell death in vitro, but activates the pro-survival nuclear factor (NF)-κB pathway. In L929 fibrosarcoma cells, TAK1 activity is required for NF-κB activity following TNFα binding, and its inhibition promotes RIPK1-dependent apoptosis or necroptosis, depending on caspase-8 activity^22,58^. Satellite cells depleted in TAK1 were recently reported to undergo spontaneous RIPK1-dependent cell death, but independently of TNFα stimulation^59^. We found in vitro that C2C12 myoblasts did not spontaneously die by pharmacological TAK1 inhibition but required TNFα treatment. In myoblasts overexpressing RIPK3, TNFα alone could trigger necroptosis in caspase-compromised conditions, no longer requiring sensitising methods to facilitate death (i.e., TAK1 inhibition) (Fig. 2i, j). In degenerating mdx muscles, the whole necroptosis machinery, including RIPK3, was upregulated and two-thirds of TA myonecrosis was RIPK3 dependent. It is tempting to speculate that the massive TNFα release at 3 weeks can represent a potent extracellular death trigger for myofibres upregulating RIPK1, RIPK3, and MLKL. However, other death ligands such as TWEAK (TNF-related weak inducer of apoptosis), interferon-γ, and Toll-like receptor-4 are released by inflammatory cells in dystrophin-deficient muscles^60^ and could possibly participate in the induction of necroptosis. Mechanical stimuli were shown to trigger necroptosis in nucleus pulposus cells^33^. Whether skeletal muscle cells can be sensitised to necroptosis by mechanical stress remains to be investigated.

In myocardial ischaemia, the depletion of RIPK3 is also associated with a reduction of inflammatory cell infiltration and fibrosis, and the generation of ROS^21,43^. ROS production affects dystrophic mouse muscles at an early stage and participates in lipid peroxidation and membrane permeability^61^. We report that the inhibition of ROS generation alleviates necroptosis in C2C12 myoblasts (Fig. 2h, i). While the mechanisms at the origin of oxidative stress in dystrophic muscles are unclear, our in vitro findings suggest that necroptosis execution could involve ROS generation in muscle cells.

Collectively, these results demonstrate that skeletal muscle tissue can undergo a programmed form of necrosis called necroptosis. More specifically, the strong peak of degeneration characterising the onset of mdx pathology is driven by RIPK3 and its depletion ameliorates myofibre survival, decreases inflammatory infiltrate, and improves muscle function.

## Methods

### Animals

Mice were bred and experimental procedures carried out at both the Biological Services Unit of the Great Ormond Street Institute of Child Health and the University College London in accordance with the Animals (Scientific Procedures) Act 1986, under Home Office Licences 70/7086 and 70/8389, and in the mouse facility of the Alfort School of Veterinary Medicine, France, registered under the number EU94-046-6. All experiments were performed following the United Kingdom, French, and European guidelines (Directive 2010/63/UE of the European Parliament and of the Council). C57BL/10 and mdx mice were purchased from the Jackson Laboratory. C57BL/6 *Ripk3*^*+/−*^ mice were kindly provided by Genentech (San Francisco, CA). Mdx*Ripk3*^*−/−*^ mice were generated by crossing Ripk3^*−/−*^ with *mdx* mice. Resulting mice were intercrossed for at least three generations to obtain *mdx*Ripk3^*+/−*^ breeders to generate progeny that were used in the experiments described, so that analysed mdx*Ripk3*^*+/+*^ (mdx phenotype) and mdx*Ripk3*^*−/−*^ mice were littermates.

### Human muscle biopsies

Human biopsies were obtained either from the MRC Centre for Neuromuscular Diseases Biobank London and were approved by the National Health Service (NHS) National Research Ethics Committee (reference number: 05/MRE12/32) or from the Henri Mondor Biological Resource Platform (registration DC-2009-930, French Ministry of Research). An informed consent was obtained from all human participants. Quadriceps biopsies from seven DMD patients were analysed and compared to six samples collected from patients for whom diagnosis involved neither muscular dystrophy nor signs of muscle degeneration by histological analysis.

### Plasmids and RNAi-mediated knockdown

C2C12 myoblasts were grown in Dulbecco's modified Eagle's medium (high glucose) supplemented with 10% foetal bovine serum, 1% GlutaMAx, and 100 U/ml penicillin–streptomycin. For RNAi studies, C2C12 cells were plated 1 day before transfection at 4 × 10^4^ cells per well of a 24-well plate in culture medium. Myoblasts were transfected with 100 nM *Ripk3* SiRNA (Dharmacon) using Lipofectamine 2000 (ThermoFisher) for 6 h before changing to fresh culture medium. Cells transfected with scrambled small interfering RNA (siRNA) were used as control. After 72 h, transfected cells were analysed by reverse transcriptase (RT) quantitative PCR or used for cell death assays.

For RIPK3 upregulation, a plasmid co-expressing the wild-type murine *Ripk3* and the GFP was used as a gift from Francis Chan (Addgene plamid #41382). A GFP-expressing plasmid (pEGFP-C2) was used as a control. C2C12 myoblasts were transfected with TurboFect transfection reagent (ThermoFisher #R0531) according to the manufacturer's instructions. Transfected cells were incubated overnight at 37 °C and on the following day, a second transfection was performed to improve the percentage of transfected cells. After 24 h, cells were GFP-sorted through fluorescence-activated cell sorting and seeded overnight before cell death assays.

### Cell death assays

Cells were seeded into 96-well plates (5000 cells per well) and pre-treated for 1 h with 1 µM TAK1 inhibitor (5Z)-7-Oxozeaenol (NP-009245, AnalytiCon Discovery GmbH), 50 µM of pan-caspase inhibitor Z-VAD.fmk (Promega), 30 µM Nec-1s (Cambridge Bioscience), 0.1–3 µM GSK’872 (Generon), 100 µM Butylated hydroxyanisole (BHA Sigma), and dimethyl sulfoxide (DMSO). Then, cells were stimulated with recombinant mouse TNFα (Peprotech) at 100 ng/ml for 3 h unless otherwise stated. Cell survival was determined by CellTiter-Glo luminescent cell viability assay (Promega) and membrane permeability by CytoTox-Glo cytotoxicity assay (Promega) according to the manufacturer’s instructions. Luminescence was read by a microplate reader Fluostar Optima (BMG Labtech).

### RNA extraction, PCR, and real-time PCR

Snap-frozen muscles were thawed and transferred in tube which contains 1.4 mM ceramic beads (Precellys, Bertin Corp, MD, USA) plus 1 ml of Trizol (Life Technologies, Saint Aubin, France) and centrifuged once at 6500 rpm for 20 s. Total RNAs were extracted using trizol according to the manufacturer’s protocol (Life Technologies, Saint Aubin, France). The quantity of RNA was determined using a Nanodrop ND-1000 spectrophotometer (Thermo Scientific, Wilmington, DE, USA). Quantitative PCR (qPCR) was designed according to the MIQE standards. qPCRs were performed on a LightCycler 480 Real-Time PCR System (Roche, Meylan, France) in a final volume of 9 µl with 0.4 µl of RT product, 0.18 µl each of forward and reverse primers (20 pmol/ml), and 4.5 µl of SYBRGreen Mastermix (Roche, Basel, Switzerland). After qPCR, the PCR products were run on a 2% agarose gel and were cloned using the Topo cloning kit (Life Technologies, Saint Aubin, France) and sequenced. Primers used in this study are the following: forward *mPsma2* 5′-AGAGCGCGGTTACAGCTTC-3′ and reverse 5′-CTCCACCTTGTGAACACTCCTT-3′, forward *mRipk3* 5′-CGGGCACACCACAGAACAT-3′, *mRipk3* and reverse 5′- GTAGCACATCCCCAGCACCAC-3′, forward *mRipk1* 5′-AGAAGAAGGGAACTATTCGC-3′ and reverse *mRipk1* 5′-TTCTATGGCCTCCACGAT-3′, and forward *mMlkl* 5′-ATCAAAGTATTCAACAACCCC-3′ and reverse *mMlkl* 5′-GCAAATCCCAAATATACGCAA-3′.

### Antibodies and western blotting

Protein extracts from mouse muscles, C2C12 myoblasts, and MEFs (strain CF-1, Millipore) were analysed using the following primary antibodies: rabbit antibody to RIPK3 clone H-43 (Santa Cruz, sc-135170, 1:300), rabbit antibody to RIPK3 (Sigma, PRS2283, 1:1000), mouse antibody to GAPDH (Abcam, 9484, 1:500). Secondary antibodies were: IRDye 680RD conjugated goat anti-rabbit IgG (LI-COR, 925-68071, 1:15,000) and IRDye 800CW conjugated goat anti-mouse IgG (LI-COR, 926-32210, 1:15,000). Detection was performed using a LI-COR Odyssey instrument (fluorescent detection) and the quantification of images was performed using ImageJ. Uncropped scans are shown in the Supplementary Figure 6.

For immunofluorescence, mouse antibody to human Laminin α2 (clone 5H2, MAB1922, 1:1000), rabbit to RIPK3 clone H-43 (Santa Cruz, sc-135170, 1:50), rabbit antibody to human phospho-MLKL (Abcam, Ab-187091, 1:80), rat antibody to CD68 (clone FA-11, 137001, 1:50), rabbit antibody to mouse pan-Laminin (Sigma, L9393, 1:1000), rabbit antibody to Collagen VI (Abcam, Ab6588, 1:150), and neonatal myosin heavy chain antibody (Clone BF34; Developmental Studies Hybridoma Bank, 1:50) were used.

### Image analysis

For the analysis of the following parameters, myonecrosis, CD68-positive cell infiltrate, NeoMHC-positive fibres, and fibrosis, transverse cryosections taken from the entire length of the TA muscle were immunostained and quantified using Metamorph software. The section with the highest area of each measured parameter (normalised to CSA) for each muscle is presented. Myonecrosis levels were determined by measuring the area corresponding to mouse IgG uptake in myofibres (and not in interstitial areas) and expressed as the percentage of the CSA. Muscle fibre minimal Feret diameter, corresponding to the minimum distance between the two parallel tangents of myofibres, was determined. Images analyses were performed with ImageJ by a specific self-developed macro that recognises muscle fibres^62^.

### Serum CK activity

Blood (50–100 µl) obtained by puncture of the mandibular vein was left for 20 min at room temperature. Serum was separated from the clot by a refrigerated centrifugation for 10 min at 2000 × *g*, and immediately stored at −80 °C. Quantification of serum CK activity was batch-performed using series of thawed sera diluted at 1:15, using the Catalyst DX Chemistry Analyzer connected to the VetLab station (IDEXX Laboratories, The USA; chemistry batches #9741 and #9608).

### Muscle strength

Grip strength was measured on male mice with a grip strength metre (BIO-GS3, Bioseb, France) according to the manufacturer’s instructions. Briefly, the four limbs were allowed to grasp the grid and mice were gently pulled backwards. Five measurements were recorded and the highest was used for analysis. Absolute value was normalised to the body weight. The hanging test was performed to assess abdominal and limb muscle fatigability. Mice were placed on a grid which was gently inverted so that mice were hanging under the grid. The latency to fall was timed and normalised to the body weight.

### Activity recording

The 10–12-week-old male mice were individually caged to form five groups of mixed mdx*Ripk3*^+/+^ and mdx*Ripk3*^−/−^ littermates. They were housed in the same rack with a 12:12 light/dark cycle, the dark onset being set at 7 pm. During 4 consecutive days at around 6:30 pm, mice were trained with an increasing moderate exercise using a treadmill (Panlab LE8710MTS, Harvard Apparatus). On day 4, they walked for 5 min at 1 cm/s followed by 3 min at 5 cm/s and then ran for 3 min at 10 cm/s, 2 min at 12 cm/s, 2 min at 14 cm/s, and ended the exercise by 2 min at 17 cm/s. Then, their nightly individual spontaneous activity was recorded from around 7 pm for 14 h using the ActivMeter system (Bioseb, France), with the activity threshold set to 2. The activity included mobile activity and immobile activity such as grooming and feeding. Inactive time included sleeping or resting with no other movements than breathing. The low speed threshold was set to 3.5 cm/s and the high speed threshold set to 7 cm/s. Activity data from 7:30 pm to 7:30 am were extracted from raw files and data recorded the night after the moderate stimulating exercise (day 4) were analysed.

## Electronic supplementary material

Supplementary Information

## Data Availability

All relevant data are available from the author upon request.

## References

[1] Gilbert RK, Hawk WA (1963). The incidence of necrosis of muscle fibers in Duchenne type muscular dystrophy. Am. J. Pathol..

[2] Moat SJ, Bradley DM, Salmon R, Clarke A, Hartley L (2013). Newborn bloodspot screening for Duchenne muscular dystrophy: 21 years experience in Wales (UK). Eur. J. Hum. Genet..

[3] Matsuda R, Nishikawa A, Tanaka H (1995). Visualization of dystrophic muscle fibers in mdx mouse by vital staining with Evans blue: evidence of apoptosis in dystrophin-deficient muscle. J. Biochem..

[4] Tidball JG, Albrecht DE, Lokensgard BE, Spencer MJ (1995). Apoptosis precedes necrosis of dystrophin-deficient muscle. J. Cell Sci..

[5] Sandri M, Carraro U (1999). Apoptosis of skeletal muscles during development and disease. Int. J. Biochem. Cell Biol..

[6] Carpenter S, Karpati G (1979). Duchenne muscular dystrophy: plasma membrane loss initiates muscle cell necrosis unless it is repaired. Brain.

[7] Rosenberg AS (2015). Immune-mediated pathology in Duchenne muscular dystrophy. Sci. Transl. Med..

[8] Wehling M, Spencer MJ, Tidball JG (2001). A nitric oxide synthase transgene ameliorates muscular dystrophy in mdx mice. J. Cell Biol..

[9] Grounds MD, Torrisi J (2004). Anti-TNFalpha (Remicade) therapy protects dystrophic skeletal muscle from necrosis. FASEB J..

[10] Hodgetts S, Radley H, Davies M, Grounds MD (2006). Reduced necrosis of dystrophic muscle by depletion of host neutrophils, or blocking TNFalpha function with Etanercept in mdx mice. Neuromuscul. Disord..

[11] Degterev A (2005). Chemical inhibitor of nonapoptotic cell death with therapeutic potential for ischemic brain injury. Nat. Chem. Biol..

[12] Vanden Berghe T, Hassannia B, Vandenabeele P (2016). An outline of necrosome triggers. Cell. Mol. Life Sci..

[13] Zhao H (2015). Role of necroptosis in the pathogenesis of solid organ injury. Cell Death Dis..

[14] He S (2009). Receptor interacting protein kinase-3 determines cellular necrotic response to TNF-alpha. Cell.

[15] Zhang DW (2009). RIP3, an energy metabolism regulator that switches TNF-induced cell death from apoptosis to necrosis. Science.

[16] Jouan-Lanhouet S (2014). Necroptosis, in vivo detection in experimental disease models. Semin. Cell Dev. Biol..

[17] Bulfield G, Siller WG, Wight PA, Moore KJ (1984). X chromosome-linked muscular dystrophy (mdx) in the mouse. Proc. Natl. Acad. Sci. USA.

[18] Grounds MD, Radley HG, Lynch GS, Nagaraju K, De Luca A (2008). Towards developing standard operating procedures for pre-clinical testing in the mdx mouse model of Duchenne muscular dystrophy. Neurobiol. Dis..

[19] Straub V, Rafael JA, Chamberlain JS, Campbell KP (1997). Animal models for muscular dystrophy show different patterns of sarcolemmal disruption. J. Cell Biol..

[20] Wang H (2014). Mixed lineage kinase domain-like protein MLKL causes necrotic membrane disruption upon phosphorylation by RIP3. Mol. Cell.

[21] Luedde M (2014). RIP3, a kinase promoting necroptotic cell death, mediates adverse remodelling after myocardial infarction. Cardiovasc. Res..

[22] Vanlangenakker N (2011). cIAP1 and TAK1 protect cells from TNF-induced necrosis by preventing RIP1/RIP3-dependent reactive oxygen species production. Cell Death Differ..

[23] Degterev A (2008). Identification of RIP1 kinase as a specific cellular target of necrostatins. Nat. Chem. Biol..

[24] Galluzzi L (2015). Essential versus accessory aspects of cell death: recommendations of the NCCD 2015. Cell Death Differ..

[25] Kaiser WJ (2013). Toll-like receptor 3-mediated necrosis via TRIF, RIP3, and MLKL. J. Biol. Chem..

[26] Goossens V, Grooten J, De Vos K, Fiers W (1995). Direct evidence for tumor necrosis factor-induced mitochondrial reactive oxygen intermediates and their involvement in cytotoxicity. Proc. Natl. Acad. Sci. USA.

[27] Lin Y (2004). Tumor necrosis factor-induced nonapoptotic cell death requires receptor-interacting protein-mediated cellular reactive oxygen species accumulation. J. Biol. Chem..

[28] Cho YS (2009). Phosphorylation-driven assembly of the RIP1-RIP3 complex regulates programmed necrosis and virus-induced inflammation. Cell.

[29] Newton K, Sun X, Dixit VM (2004). Kinase RIP3 is dispensable for normal NF-kappa Bs, signaling by the B-cell and T-cell receptors, tumor necrosis factor receptor 1, and Toll-like receptors 2 and 4. Mol. Cell. Biol..

[30] Galluzzi L (2009). Guidelines for the use and interpretation of assays for monitoring cell death in higher eukaryotes. Cell Death Differ..

[31] Tidball JG (2017). Regulation of muscle growth and regeneration by the immune system. Nat. Rev. Immunol..

[32] Vanden Berghe T, Linkermann A, Jouan-Lanhouet S, Walczak H, Vandenabeele P (2014). Regulated necrosis: the expanding network of non-apoptotic cell death pathways. Nat. Rev. Mol. Cell Biol..

[33] Chen S (2017). RIPK1/RIPK3/MLKL-mediated necroptosis contributes to compression-induced rat nucleus pulposus cells death. Apoptosis.

[34] Li D (2017). RIPK1-RIPK3-MLKL-dependent necrosis promotes the aging of mouse male reproductive system. Elife.

[35] Siempos II (2018). RIPK3 mediates pathogenesis of experimental ventilator-induced lung injury. JCI Insight.

[36] Janssen I, Heymsfield SB, Wang ZM, Ross R (2000). Skeletal muscle mass and distribution in 468 men and women aged 18-88 yr. J. Appl. Physiol. (1985).

[37] Sandri M, Minetti C, Pedemonte M, Carraro U (1998). Apoptotic myonuclei in human Duchenne muscular dystrophy. Lab. Invest..

[38] Gunther C (2016). The pseudokinase MLKL mediates programmed hepatocellular necrosis independently of RIPK3 during hepatitis. J. Clin. Invest..

[39] Sandri M (2001). Caspase 3 expression correlates with skeletal muscle apoptosis in Duchenne and facioscapulo human muscular dystrophy. A potential target for pharmacological treatment?. J. Neuropathol. Exp. Neurol..

[40] Bell RAV, Megeney LA (2017). Evolution of caspase-mediated cell death and differentiation: twins separated at birth. Cell Death Differ..

[41] Fernando P, Kelly JF, Balazsi K, Slack RS, Megeney LA (2002). Caspase 3 activity is required for skeletal muscle differentiation. Proc. Natl. Acad. Sci. USA.

[42] Trichonas G (2010). Receptor interacting protein kinases mediate retinal detachment-induced photoreceptor necrosis and compensate for inhibition of apoptosis. Proc. Natl. Acad. Sci. USA.

[43] Zhang T (2016). CaMKII is a RIP3 substrate mediating ischemia- and oxidative stress-induced myocardial necroptosis. Nat. Med..

[44] Huang JF (2013). Differential neuronal expression of receptor interacting protein 3 in rat retina: involvement in ischemic stress response. BMC Neurosci..

[45] Afonso MB (2016). Activation of necroptosis in human and experimental cholestasis. Cell Death Dis..

[46] Ofengeim D (2015). Activation of necroptosis in multiple sclerosis. Cell Rep..

[47] Stedman HH (1991). The mdx mouse diaphragm reproduces the degenerative changes of Duchenne muscular dystrophy. Nature.

[48] Serrano AL, Munoz-Canoves P (2010). Regulation and dysregulation of fibrosis in skeletal muscle. Exp. Cell Res..

[49] Beastrom N (2011). mdx((5)cv) mice manifest more severe muscle dysfunction and diaphragm force deficits than do mdx Mice. Am. J. Pathol..

[50] Najjar M (2016). RIPK1 and RIPK3 kinases promote cell-death-independent inflammation by Toll-like receptor 4. Immunity.

[51] Kearney CJ (2015). Necroptosis suppresses inflammation via termination of TNF- or LPS-induced cytokine and chemokine production. Cell Death Differ..

[52] Kim EY (2017). Correlation of serum creatine kinase level with pulmonary function in Duchenne muscular dystrophy. Ann. Rehabil. Med..

[53] Allen DG, Whitehead NP (2011). Duchenne muscular dystrophy--what causes the increased membrane permeability in skeletal muscle?. Int. J. Biochem. Cell Biol..

[54] Arnold L (2007). Inflammatory monocytes recruited after skeletal muscle injury switch into antiinflammatory macrophages to support myogenesis. J. Exp. Med..

[55] Bencze M (2012). Proinflammatory macrophages enhance the regenerative capacity of human myoblasts by modifying their kinetics of proliferation and differentiation. Mol. Ther..

[56] Leite PE (2010). Nicotinic acetylcholine receptor activation reduces skeletal muscle inflammation of mdx mice. J. Neuroimmunol..

[57] Lemos DR (2015). Nilotinib reduces muscle fibrosis in chronic muscle injury by promoting TNF-mediated apoptosis of fibro/adipogenic progenitors. Nat. Med..

[58] Dondelinger Y (2013). RIPK3 contributes to TNFR1-mediated RIPK1 kinase-dependent apoptosis in conditions of cIAP1/2 depletion or TAK1 kinase inhibition. Cell Death Differ..

[59] Ogura Y (2015). TAK1 modulates satellite stem cell homeostasis and skeletal muscle repair. Nat. Commun..

[60] De Paepe B, De Bleecker JL (2013). Cytokines and chemokines as regulators of skeletal muscle inflammation: presenting the case of Duchenne muscular dystrophy. Mediat. Inflamm..

[61] Whitehead NP, Pham C, Gervasio OL, Allen DG (2008). N-Acetylcysteine ameliorates skeletal muscle pathophysiology in mdx mice. J. Physiol..

[62] Hourde C (2013). Protective effect of female gender-related factors on muscle force-generating capacity and fragility in the dystrophic mdx mouse. Muscle Nerve.

