# Publisher Correction: Necroptosis mediates myofibre death in dystrophin-deficient mice

**DOI:** 10.1038/s41467-018-06636-w

**Published:** 2018-10-02

**Authors:** Jennifer E. Morgan, Alexandre Prola, Virginie Mariot, Veronica Pini, Jinhong Meng, Christophe Hourde, Julie Dumonceaux, Francesco Conti, Frederic Relaix, Francois-Jerôme Authier, Laurent Tiret, Francesco Muntoni, Maximilien Bencze

**Affiliations:** 1 The Dubowitz Neuromuscular Centre, Molecular Neurosciences Section, Developmental Neurosciences Programme, UCL Great Ormond Street Institute of Child Health, London, WC1N 1EH UK; 2 U955-IMRB, Team 10, Biology of the Neuromuscular System, Inserm, UPEC, ENVA, EFS, Créteil 94000, France; 3 NIHR Biomedical Research Centre, University College London, Great Ormond Street Institute of Child Health and Great Ormond Street Hospital NHS Trust, 30 Guilford Street, London, WC1N 1EH UK; 4 Inter-University Laboratory of Human Movement Biology (LIBM)-EA7424, Université Savoie Mont Blanc, Campus Scientifique Technolac, 73376 Le Bourget du Lac Cedex, France; 5 Nord/Est/Ile-de-France Reference Centre for Neuromuscular Diseases, Henri Mondor University Hospital (APHP), 94000 Créteil, France

Correction to: *Nature Communications* 10.1038/s41467-018-06057-9; published online 07 September 2018

The original version of this article contained an error in Fig. 3. In panel c, the labels ‘mdx’ and ‘mdx Ripk3-/-‘ were inadvertently inverted. This has now been corrected in the PDF and HTML versions of the article.

