# Supplementary material for: Necroptosis mediates myofibre death in dystrophin-deficient mice
Source: Nat Commun. 2018 Sep 7;9:3655. doi: 10.1038/s41467-018-06057-9 (PMC6128848; doi:10.1038/s41467-018-06057-9)
Supplement: Supplementary file 1 — Supplementary Information [file 41467_2018_6057_MOESM1_ESM.pdf]

Necroptosis mediates myofibre death in dystrophin-deficient mice

Supplementary Figures (1-6)

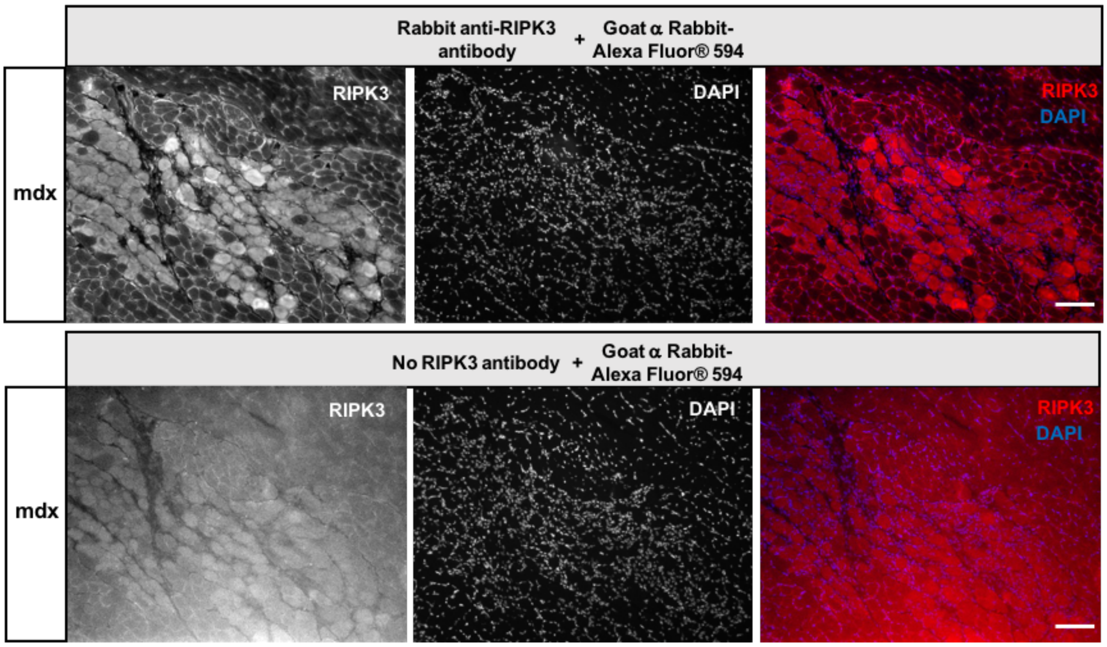

Supplementary Figure 1: RIPK3 immunoreactivity in *mdx* muscles.

In order to verify that RIPK3 immunoreactivity was due to the primary antibody binding, serial sections of *mdx gastrocnemius* were stained with (upper panels), or without (lower panels) primary antibody raised in rabbit and directed against RIPK3 antigen. Secondary goat anti-rabbit was applied to both serial sections and images were acquired with identical settings (including exposure time) of the same muscle region. Scale bar, 100μm.

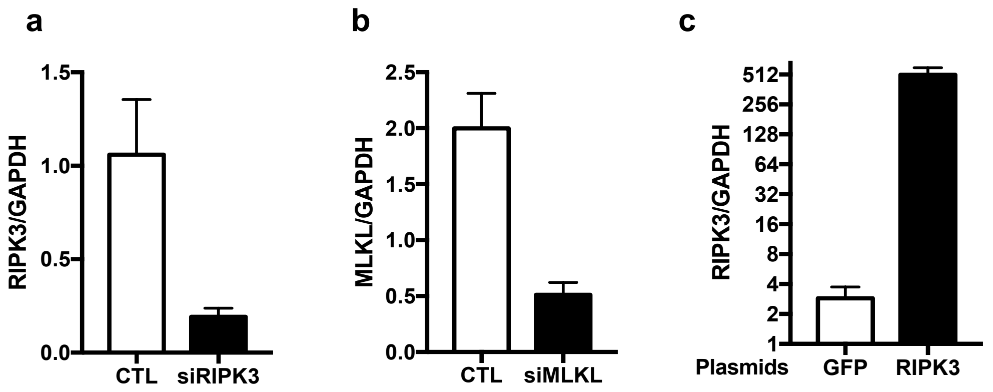

Supplementary Figure 2: RIPK3 and MLKL expression in transfected C2C12 cells.

Ripk3 or *Mlkl* were knocked down using specific RNA interfering sequences. 48 hours after cell transfection, RNA levels of *Ripk3* **(a)** and *Mlkl* **(b)** were retrotranscribed, assessed by quantitative PCR and normalised to *Gapdh* levels. **(c)** C2C12 cells were transfected with a *GFP*-plasmid or a *GFP*-tagged *Ripk3* plasmids and *Ripk3* RNA level was examined. Representative of two independent experiments (three replicates per group). Data shown as the mean ± SEM.

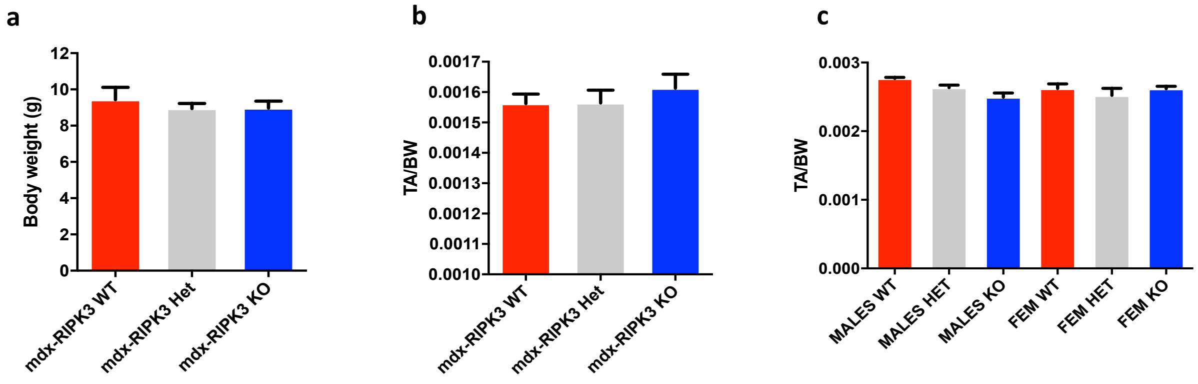

Supplementary Figure 3: Body and TA weights are not affected by RIPK3 deficiency.

(a) Body weight (BW) of 3-week-old mice of different genotypes. Females and males were mixed. n = 4 mdx*Ripk3*+/+, n = 11 mdx*Ripk3*+/-, n = 10 mdx*Ripk3*-/- mice. **(b)** TA weight was measured in 3-week-old mice and normalised to the mouse body weight (TA/BW). n = 8 mdx*Ripk3*+/+, n = 22 mdx*Ripk3*+/-, n = 18 mdx*Ripk3*-/- TA. **(c)** TA normalised to body weight. Males: n = 2 mdx*Ripk3*+/+, n = 18 mdx*Ripk3*+/-, n = 10 mdx*Ripk3*-/- mice. Females: n = 8 mdx*Ripk3*+/+, n = 8 mdx*Ripk3*+/-, n = 8 mdx*Ripk3*-/- mice. Data shown as the mean ± SEM.

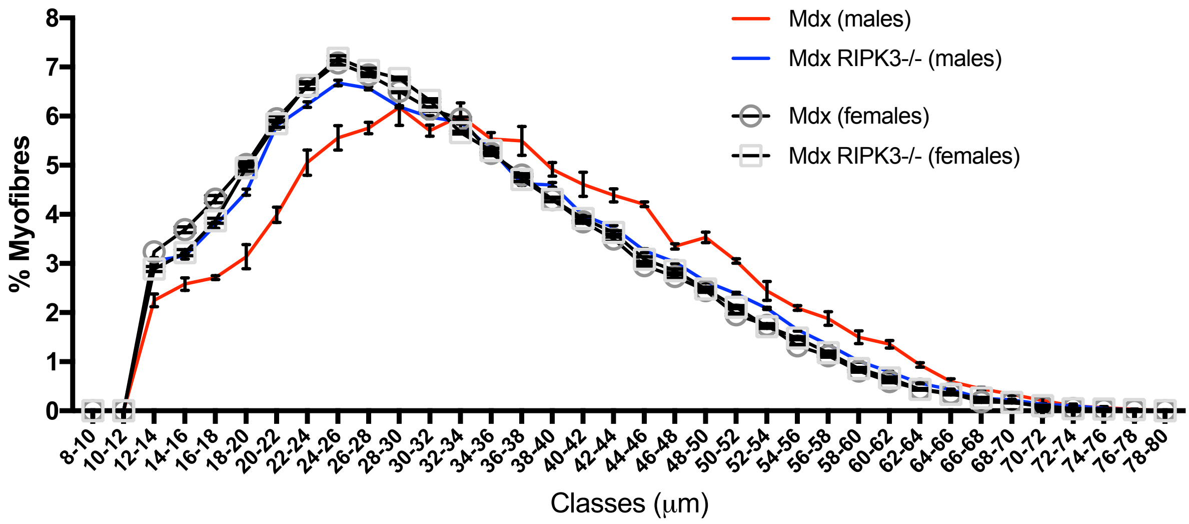

Supplementary Figure 4: RIPK3 deficiency affects fibres size in mdx males

Fibre size distribution in mdx and mdx*Ripk3*-/- TA at 9 weeks. Data expressed as mean percentage of fibres size (min. feret) (females: n = 7 mdx, n = 8 mdx*Ripk3*-/-; males n = 2 mdx, n = 10 mdx*Ripk3*-/-).

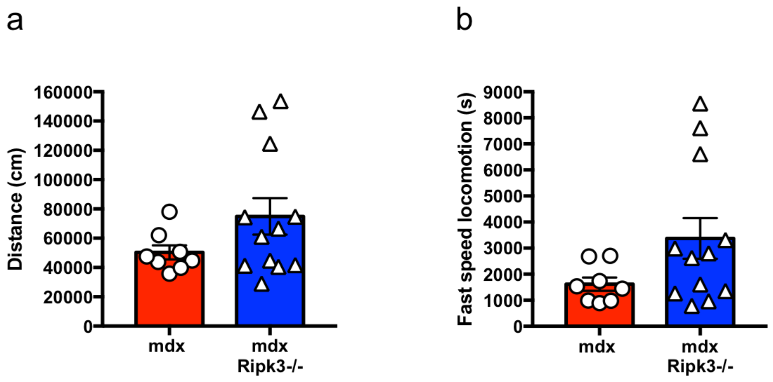

Supplementary Figure 5: Locomotion parameters in mdx and mdx*Ripk3*$^{-/-}$ mice

(a) Distance covered by 3-months old mice in 12 hours. **(b)** Duration of fast speed locomotion in 12 hours. Data expressed as the mean ± SEM (n = 8 mdx, n = 12 mdx*Ripk3-/-*).

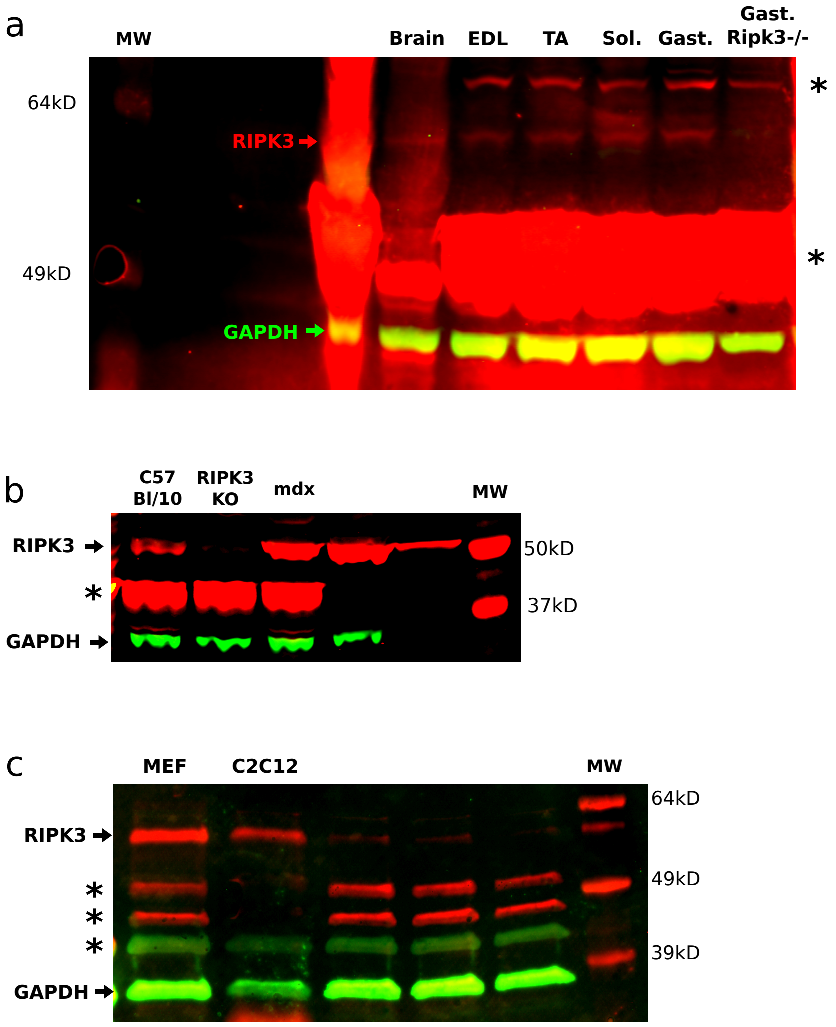

Supplementary Figure 6: Uncropped scans of blots

Uncropped scans corresponding to Figure 1a **(a)**, Figure 1c **(b)** and Figure 2a **(c)**. Detection was performed using a LI-COR Odyssey instrument (fluorescent detection). RIPK3 was detected using an IRDye 680RD conjugated goat anti-rabbit IgG antibody (red) and GAPDH using an IRDye 800CW conjugated goat anti-mouse IgG (green). Unspecific bands are indicated by *. MW: Molecular weight.
